# Directly targeting PRDM16 in thermogenic adipose tissue to treat obesity and its related metabolic diseases

**DOI:** 10.3389/fendo.2024.1458848

**Published:** 2024-09-16

**Authors:** Liufeng Mao, Jinli Lu, Yunliang Hou, Tao Nie

**Affiliations:** ^1^ The First Affiliated Hospital, Guangdong Pharmaceutical University, Guangzhou, China; ^2^ School of Basic Medicine, Hubei University of Arts and Science, Xiangyang, China

**Keywords:** obesity, brown adipocyte, beige adipocyte, PRDM16, UCP1

## Abstract

Obesity is increasing globally and is closely associated with a range of metabolic disorders, including metabolic associated fatty liver disease, diabetes, and cardiovascular diseases. An effective strategy to combat obesity involves stimulating brown and beige adipocyte thermogenesis, which significantly enhances energy expenditure. Recent research has underscored the vital role of PRDM16 in the development and functionality of thermogenic adipocytes. Consequently, PRDM16 has been identified as a potential therapeutic target for obesity and its related metabolic disorders. This review comprehensively examines various studies that focus on combating obesity by directly targeting PRDM16 in adipose tissue.

## 1 Introduction

Obesity is becoming a global epidemic and is closely associated with various chronic diseases, such as type 2 diabetes, cardiovascular disorders, and hypertension (1). In recent years, non-shivering thermogenesis in adipose tissues has emerged as a promising therapeutic target for obesity and its related comorbidities. Adipocyte thermogenesis occurs when white adipocytes or beige precursor cells transform into beige adipocytes in white adipose tissue (WAT), and brown adipocytes are activated in brown adipose tissue (BAT). This process involves increased expression of thermogenic genes and proteins, such as uncoupling protein 1 (UCP1), which is highly expressed in beige and brown adipocytes (thermogenic adipocytes) and can produce heat by dissipating the proton motive force without generating adenosine triphosphate (ATP). UCP1 is the most extensively studied mediator of thermogenesis. However, a variety of additional mechanisms have also been identified to promote thermogenic energy expenditure in adipocytes. These include fatty acid oxidation, creatine phosphorylation, and calcium cycling (2). These changes enhance the adipocytes’ capacity to produce heat, thereby increasing energy expenditure and promoting weight loss. Numerous studies have demonstrated that enhancing adipocyte thermogenesis can reduce fat mass and improve metabolic health (3–6). It is noted that human brown adipose tissue is not only present in the interscapular region of infants but is also identified in various anatomical parts of adults (7, 8). Cold exposure or adrenergic receptor agonists can activate brown adipocytes in humans (9–12). The adrenergic stress response to prolonged cold exposure has been observed to induce browning of subcutaneous white adipose tissue (sWAT) in rodents. In contrast, in humans, ten days of cold exposure, which resulted in the BAT activation, but did not lead to the browning of sWAT (13). Interestingly, burn injury can result in prolonged elevations of circulating norepinephrine levels, which in turn leads to an increase in UCP1-positive beige adipocytes within the sWAT. This observation has been linked to the severe adrenergic stress response and the browning of sWAT in humans (14). Furthermore, BAT secretes a multitude of cytokines that facilitate communication with other organ systems, potentially contributing to the systemic metabolic effects (15).

The mechanisms underlying adipocyte thermogenesis involve transcriptional regulation, environmental cues, and signaling pathways. The key transcription factor PRDM16 plays a vital role in adipocyte thermogenesis. The PRDM protein family consists of 17 members that structurally contain a conserved N-terminal PR (PRDI-BF1 and RIZ1 homology) domain, which is similar to the SET (suppressor of variegation, enhancer of zeste, and trithorax) domain found in many histone lysine methyltransferases (HMTs). The PRDM proteins also contain a variable number of zinc finger domains, which directly bind with DNA. PRDM proteins are involved in various cellular processes, which include cell fate decision (16, 17). The *PRDM16* gene, which was also named MDS1/EVI1-like gene 1 (*MEL1*) by Mochizuki et al., was first discovered in patients with acute myeloid leukemia and the myelodysplastic syndrome (18). The human *PRDM16* gene (located on chromosome 11p36.32) and the mouse *Prdm16* gene (located on chromosome 4qE2) contain 17 exons and encode a protein with a positive regulatory (PR) domain, an inhibitory domain (RD), two zinc finger DNA binding domains (ZF1 and ZF2), and an acidic domain (AD) (19, 20).

Recent studies have provided comprehensive information about the role of PRDM16 in adipose tissue. This review summarizes the function and regulation of PRDM16 in adipose tissue and proposes the application of this transcription factor as a potential target for treating obesity and its related metabolic disorders. Additionally, the review includes a summary of references that report proteins directly interacting with PRDM16, as well as factors influencing the expression of *Prdm16* mRNA and protein. These studies have also elucidated the physiological roles of these genes in the obesity and its related metabolic diseases.

## 2 Function of PRDM16 in adipose tissue

The physiological role of PRDM16 in adipose tissue, along with its mechanism of action, a topic of significant interest in the field of endocrinology and metabolism, is summarized below:

### 2.1 Direct function in adipose tissue

#### 2.1.1 Inducing brown adipocyte characteristics in white adipocytes

PRDM16 is significantly enriched in brown adipocytes as compared to white adipocytes. When *Prdm16* is overexpressed in white adipocytes, a robust brown fat phenotype is activated, leading to a remarkable increase in uncoupled respiration. Conversely, when *Prdm16* is knocked down using shRNA in brown adipocytes, the brown characteristics are significantly reduced. Mechanistically, PRDM16 directly binds to the promoters of *Ucp1* and PPAR gamma coactivator 1 alpha (*Pgc1α*), key regulators of brown adipocyte thermogenic function. Additionally, PRDM16 interacts with the PGC1α transcriptional coactivator to modulate brown fat determination and function (21).

White fat depots include subcutaneous adipose tissue and several intra-abdominal adipose tissues, including mesenteric adipose tissue (22). Adiposity is a major risk factor for metabolic diseases in both humans and rodents (23–25). In contrast, the expansion of subcutaneous adipose tissue has been suggested to promote insulin sensitivity in both rodents and humans (26–28). The expression levels of *Prdm16* are higher in subcutaneous white adipocytes than in other abdominal white adipocytes in mice. Transgenic expression of *Prdm16* in white fat, under the -5kb *aP2* promoter/enhancer, strongly promoted the development of beige adipocytes in subcutaneous adipose tissues but not in epididymal adipose tissues (29). When mice with overexpressed *Prdm16* were fed on high-fat diets, increased energy expenditure, reduced body weight gain, and improved glucose tolerance were observed (29). In adipose-specific *Prdm16* knockout mice (Adipo-*Prdm16* KO), which were generated by *Prdm16* ^lox/lox^ and Adiponectin-cre mice, minimal effects on classical brown fat were noted. However, beige adipocyte function was markedly inhibited in subcutaneous white adipose tissue. When fed on high-fat diets, Adipo-*Prdm16* KO developed obesity, severe insulin resistance, and hepatic steatosis. The subcutaneous adipose tissue in Adipo-*Prdm16* KO mice acquired many key properties of visceral fat, including decreased thermogenic gene expression, increased inflammatory gene expression, and macrophage accumulation (30). Furthermore, adipocyte progenitor cells derived from human white adipose tissue demonstrate beige adipocyte characteristics upon differentiation into white adipocytes, as revealed through single-cell RNA sequencing analysis. These cells exhibit a pronounced enrichment of markers traditionally associated with beige adipocytes, including *PGC1α*, *PRDM16*, and *UCP1* (31). These findings indicate that PRDM16 is essential for the formation of beige adipocytes and enhances the health effects of subcutaneous adipose tissue.

#### 2.1.2 Controlling the switch of myoblasts into brown adipocytes

Although white and brown adipocytes both contain lipid droplets and display some morphological similarities, their developmental origins differ markedly. Brown adipocytes share the *Myf5*-positive precursor with myoblasts. PRDM16 is the key effector that controls the bidirectional cell fate switch between skeletal myoblasts and brown fat cells. Loss of *Prdm16* in brown preadipocytes blocks brown adipogenesis and promotes muscle differentiation. Conversely, ectopic expression of *Prdm16* in myoblasts induces their differentiation into brown adipocytes. Studies have shown that the adipogenic conversion of myoblasts to brown adipocytes by PRDM16 depends on the presence of rosiglitazone, a specific agonist for peroxisome proliferator-activated receptor gamma (PPARγ). PPARγ is the master regulator of adipogenic differentiation (32, 33). PPARγ alone can convert myogenic cells into adipocytes, but not into brown adipocytes; the latter requires PRDM16 expression. PRDM16 binds to PPARγ, thereby activating its transcriptional function and driving the differentiation of myoblasts into brown adipocytes (34). In a single-cell RNA sequencing analysis of human perivascular adipose tissue (PVAT), a brown-like fat depot, Angueira et al. identified that preadipocytes express the adipogenic gene *PPARγ*, while mature adipocytes express thermogenic genes *UCP1* and *PRDM16* (35).

The global homozygous *Prdm16*-deficient mouse died at birth. The putative BAT from the *Prdm16* knockout (KO) mice at late-stage embryos (E17) exhibited substantially larger lipid droplets, accompanied by lower brown adipocyte-selective gene expression and higher skeletal myogenic gene expression. Interestingly, embryonic BAT development in mice that selectively lacked *Prdm16* in the *Myf5* lineage was normal, with no observable discrepancies when compared with control mice. In contrast to juvenile mice, Myf5-*Prdm16* KO mice that were more than 6 months old exhibited profound morphological whitening in BAT. However, tissue temperature dramatically dropped or oxygen consumption was marginally raised in Myf5-*Prdm16* KO mice upon cold exposure or norepinephrine (NE) stimulation. *Prdm3*, closely related to *Prdm16* based on sequence and structure, compensates for the ablation of *Prdm16* in embryonic brown fat development. *Prdm3* not only regulates white adipocyte differentiation (36), but also could induce the expression of *Ucp1* and *Pgc1α*. The double KO of *Prdm3* and *Prdm16* in the *Myf5* lineage caused a more obvious white fat phenotype than in WT and *Prdm16* KO mice (37). The above studies suggest that PRDM16 is crucial for determining thermogenic adipocyte fate by interacting with PGC1α and PPARγ together.

### 2.2 Indirect function in adipose tissue

**Figure 1:**
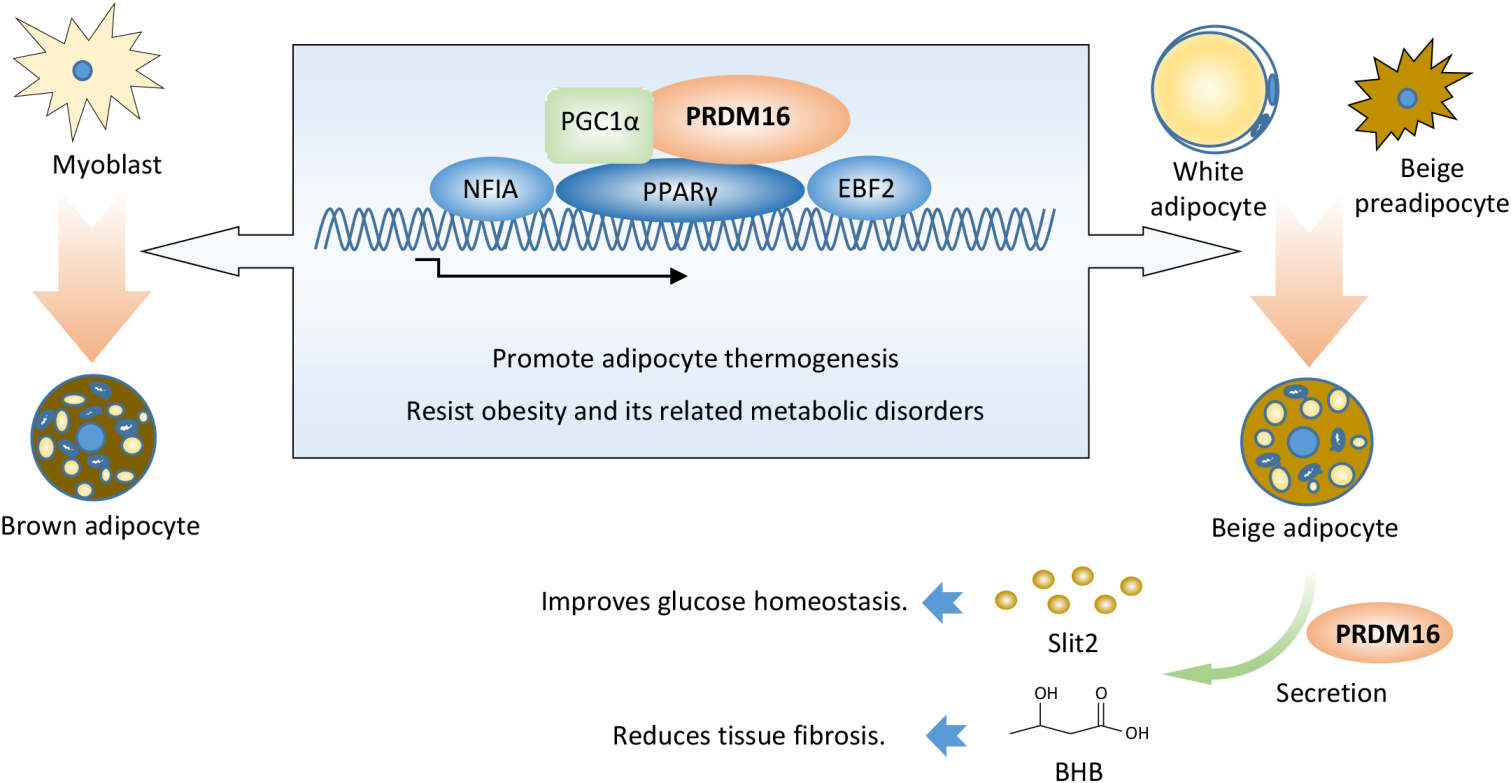
PRDM16 promotes thermogenic adipocyte function and formation. PRDM16 coordinates with PPARγ, PGC1α, EBF2, and NFIA to determine thermogenic adipocyte fate. PRDM16 regulates the expression of other genes, thereby indirectly modulating adipose tissue function. PRDM16 promotes the secretion of SLIT2 protein in beige adipocytes, thereby inducing adipocyte thermogenesis and improving insulin sensitivity. PRDM16 induces BHB secretion, which aids the differentiation of beige adipocytes and represses adipose fibrosis. BHB: β-hydroxybutyrate.

PRDM16 not only directly regulates the formation and function of brown and beige fat but also indirectly modulates the expression of other genes to control obesity and its related metabolic diseases (**Figure 1**).

Slit2, a 180 kDa member of the Slit extracellular protein family, was identified as a secreted factor of beige adipocytes from aP2-*Prdm16* transgenic mice. Full-length Slit2 is cleaved to generate several smaller fragments. The C-terminal fragment of Slit2 (Slit2-C) is an active thermogenic moiety. Slit2-C induces the thermogenesis of brown and beige adipocytes by activating the PKA signaling pathway. It promotes adipose thermogenesis, augments energy expenditure, and improves glucose homeostasis *in vivo*. However, the protease that generates Slit2-C from full-length Slit2, along with the effective surface receptor of Slit2-C, remains unknown (38).

Aging impairs beige adipogenesis and promotes fibrogenesis (39, 40). Genetic loss of *Prdm16* mimics the effects of aging, whereas increasing PRDM16 in aged mice reduces fibrosis and restores beige adipogenic potential. LC-MS analysis revealed that PRDM16-driven fatty acid oxidation produces a paracrine factor, beta-hydroxybutyrate (BHB). BHB locally or selectively accumulates in the inguinal WAT (iWAT) of young animals, thereby suppressing myofibrogenesis and stimulating beige adipogenic competency in precursor cells. BHB catabolism is mediated by BDH1 and is required for beige fat differentiation. BHB supplementation in aged animals reduced adipose fibrosis and promoted beige fat formation. However, it is still unclear whether BHB would have antifibrotic or pro-beige adipogenic effects in human adipose tissue (41).

## 3 Proteins resist obesity and its related metabolic disorders by directly interacting with PRDM16

**Figure 2:**
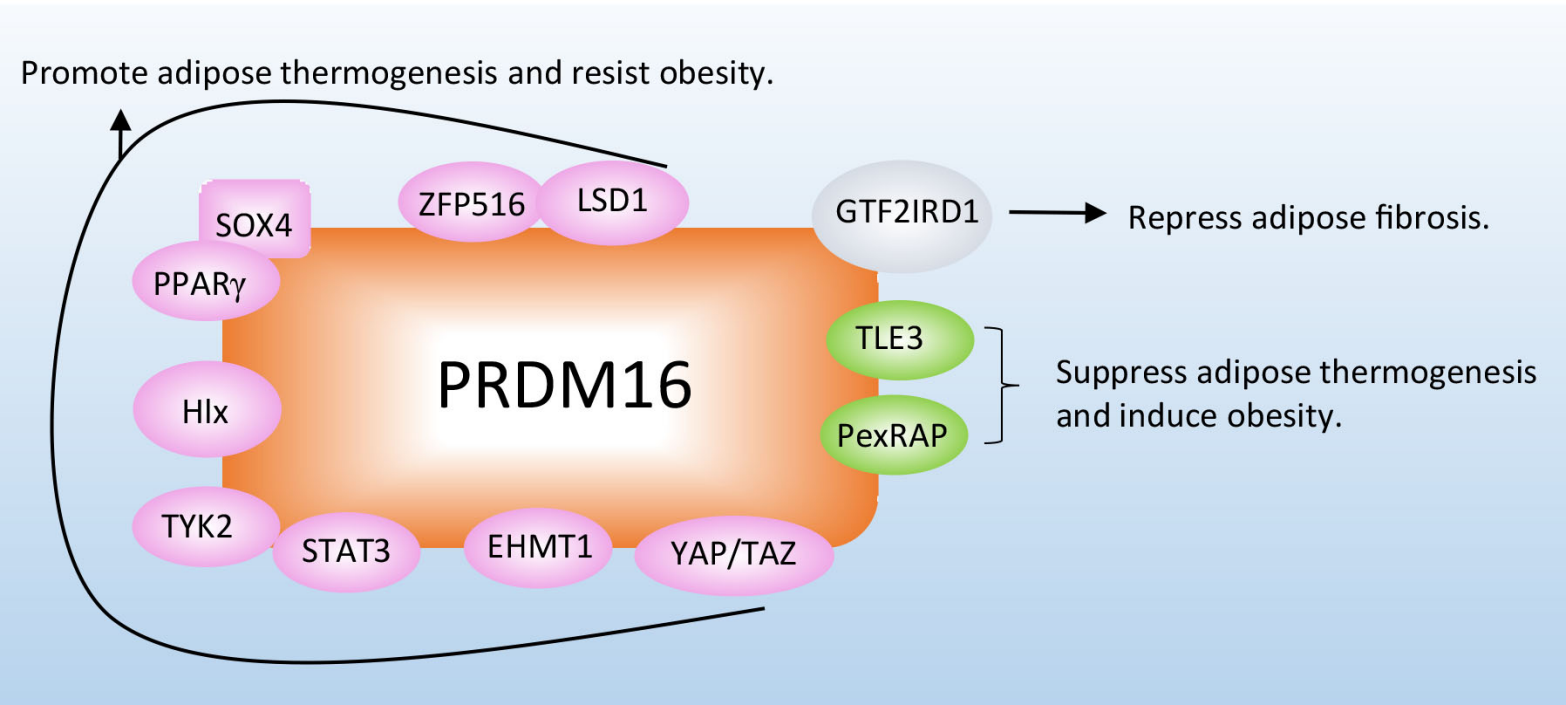
Proteins combat obesity and its related metabolic disorders by directly interacting with PRDM16. The proteins interact with PRDM16 to regulate adipocyte thermogenesis, depending on different mechanisms. More detailed information about these proteins is listed in **Table 1**.

Different types of proteins have been reported to regulate adipocyte thermogenesis or fibrosis to combat adiposity in adipose tissue by directly interacting with the PRDM16 protein (**Figure 2**; **Table 1**).

**Table 1:** Proteins that directly interact with PRDM16.

Protein name	Cellular Function	Physiological Fucntion	Human study	Reference
EHMT1	Stabilize the PRDM16 protein and control brown adipose cell fate.	Adipose-specific knockout of *Ehmt1* leads to obesity and systemic insulin resistance.	40–50% of patients with *EHMT1* mutations develop obesity.	(52, 53)
TYK2/STAT3	Binds to PRDM16 and enhances its stability to determine brown fat lineage.	*Tyk2* knockout mice developed obesity and displayed insulin resistance.	*TYK2* levels are decreased in obese humans.	(60, 61)
ZFP516	Interact with PRDM16 to activate the *Ucp1* promoter in beige and brown adipocyte.	Overexpression of *Zfp516* in adipose tissue prevents obesity and improves glucose tolerance.	None	(62, 64)
LSD1	Associates with PRDM16 to repress expression of white fat-selective genes.	Adipose-specific ablation of *Lsd1* impaires mitochondrial function and increases fat deposition.	None.	(64, 65)
Hlx	Interacts with PRDM16 to control thermogenic gene expression and mitochondrial biogenesis.	Transgenic expression of *Hlx* improves glucose homeostasis and prevents obesity and hepatic steatosis.	Human ortholog of Hlx induced *UCP1* expression and mitochondrial biogenesis in human adipocytes.	(67)
SOX4	Recruits PRDM16 to PPARγ to elevate the expression of thermogenic genes.	Adipose specific knockout of *Sox4* mice develop obesity with severe hepatic steatosis, insulin resistance, and inflammation.	None	(68)
YAP/TAZ	Inhibit sympathetic innervation of beige fat by repressing the neurotropic factor S100B.	*Yap/Taz* loss in adipocytes counteracts age-associated obesity and insulin resistance.	S100B was greatly decreased while TAZ was increased in obese human.	(74)
GTF2IRD1	Forms a complex with PRDM16 and EHMT1 to represses adipose tissue fibrosis in an UCP1 independent manner.	Adipocyte selective expression of *GTF2IRD*1 represses adipose tissue fibrosis and improves systemic glucose homeostasis.	*GTF2IRD1* expression inversely correlates with subcutaneous WAT fibrosis in humans.	(75)
TLE3	Disrupts the interaction between PPARγ and PRDM16 to suppress brown-selective genes expression.	Ad-*Tle3* KO improves thermogenic response, but not affect body weight and adipose tissue mass, possible due to a trend toward decreased activity.	Subcutaneous adipose tissue *TLE3* is increased in type 2 diabetes and decreased in bariatric surgery-induced weight loss.	(77, 78)
PexRAP	Interacts with PPARγ and PRDM16 and inhibit brown adipocyte gene expression and white adipocyte browning.	Inducible *PexRAP* KO significantly reduces adiposity.	None	(80)

### 3.1 Sequence-specific transcriptional factors to determine cell fate of thermogenic adipocytes

Although PRDM16 contains a DNA-binding domain, this domain is dispensable for PRDM16’s role in determining the fate of thermogenic adipocytes. The R998Q mutant allele of PRDM16 completely loses its DNA-binding ability but still retains the capability to interact with PPARγ to activate the brown fat gene program (21). PRDM16 indirectly binds to the genome via transcription factors that recognize specific motifs and directly binds to DNA, including PPARγ. Although PPARγ can interact with PRDM16 to convert myoblasts into brown adipocytes, its expression levels are quite low in primary and immortalized myoblasts. The Bruce M. Spiegelman group analyzed proteomic transcriptional complexes formed with wild-type (WT) PRDM16 or different mutant alleles of PRDM16 and found that C/EBP-β, prior to PPARγ, cooperates with PRDM16 to initiate the conversion of myoblasts to brown adipocytes. Forced expression of PRDM16 and C/EBP-β is sufficient to induce a fully functional brown fat program in naive fibroblastic cells, including in murine and human skin fibroblasts (42).

Nuclear factor I-A (NFIA) is regarded as a transcriptional regulator of brown adipocytes, as identified through genome-wide open chromatin analysis. NFIA and PPARγ co-localize at brown adipocyte-specific enhancers to facilitate PPARγ binding to these enhancers and drive active transcription. Overexpression of *Nfia* in myoblasts promotes brown adipocyte differentiation while inhibiting myogenic differentiation. Conversely, *Nfia* KO impairs the expression of brown fat-specific genes and elevates the expression of muscle genes. Interestingly, NFIA does not physically bind to PRDM16, and PRDM16 is dispensable for NFIA’s effects. NFIA and PRDM16 operate in parallel to each other (43). Additionally, NFIA in adipocytes can down-regulate pro-inflammatory cytokine genes to ameliorate adipose tissue inflammation by binding to the regulatory region of the *Ccl2* gene. CCL2 expression is negatively correlated with NFIA expression in human adipose tissue (44).

Early B-cell factor 2 *(Ebf2)* is also a marker gene of brown and beige adipogenic precursor cells. *Ebf2*-expressing precursor cells from brown adipose tissue and white adipose tissue differentiate into brown and beige adipocytes, respectively (45). PPARγ genome-wide Chromatin immunoprecipitation (ChIP)-Seq revealed that the EBF DNA binding motif was highly enriched in brown adipose-specific PPARγ binding sites. EBF2 increases PPARγ binding at brown-specific sites. Conversely, it significantly reduces PPARγ binding at white-specific sites. It is important to note that EBF2 is recruited to the *Prdm16* locus before PPARγ. When overexpressed in myoblasts or white adipose stromal cells, EBF2 recruits PPARγ to its brown-selective binding sites to drive a brown fat-specific differentiation program. *Ebf2*-deficient mice typically die soon after birth. An analysis of BAT in late-stage mouse embryos showed that *Ebf2* KO resulted in the loss of brown-specific characteristics and thermogenic capacity in brown adipocytes and tissue (46). Consistently, EBF2 physically interacts with the chromatin remodeler BRG1 and the BAF chromatin remodeling complex component DPF3 to activate brown fat identity genes (47). Furthermore, transgenic expression of *Ebf2 i*n adipose tissue under the aP2 promoter/enhancer powerfully stimulates beige fat development and protects animals against obesity (48). In human white adipose tissue, a subtype of adipocytes with potential thermogenic properties has been identified, characterized by the expression of thermogenic markers *PPARGC1A* and *EBF2* through single-cell RNA sequencing analysis (49). While a direct interaction between PRDM16 and EBF2 has not been reported, multiple independent studies have confirmed that these two factors co-localize at the brown adipocyte-specific enhancers, suggesting that PRDM16 could potentially directly interact with EBF2 to form a substantial transcriptional complex.

### 3.2 Co-factors and histone modification enzymes to determine cell fate of thermogenic adipocytes

High-resolution liquid chromatography coupled with tandem mass spectrometry (LC-MS/MS) showed that EHMT1, which is the only methyltransferase with enzymatic activity on H3K9 mono- or di-methylation, was in the PRDM16 complex (50). The PR-domain of PRDM16 shares high homology with methyltransferase SET domains (51). The PRDM16 complex purified from brown adipocytes expressing the PR mutant exhibited significant methyltransferase activity on H3, due to the presence of EHMT1. Consistent with this, both WT PRDM16 and the PR mutant could convert myoblasts into brown adipocytes. Loss of *Ehmt1* in brown adipocytes reduces brown fat characteristics. Conversely, overexpression of *Ehmt1* positively promotes the expression of BAT-selective genes by stabilizing the PRDM16 protein. Adipose-specific ablation of *Ehmt1* leads to a significant reduction in BAT-mediated adaptive thermogenesis and the development of severe obesity and systemic insulin resistance. Notably, patients with *EHMT1* mutations develop obesity, possibly due to impaired adaptive thermogenesis. Moreover, activation of classical brown adipocytes in the adult human perirenal depot required PRDM16-EHMT1 complex expression (52, 53).

The Mediator complex, an evolutionarily conserved multiprotein entity, is integral to transcriptional regulation. It facilitates this process by directly interacting with RNA Polymerase II and coordinating the activities of various co-activators and co-repressors at the chromatin level (54). In adipocytes, the MED1 subunit of the Mediator complex modulates the function of key transcription factors, including PPARγ, PGC-1α, and C/EBPβ, which also interact with PRDM16 (55, 56).

PRDM16 is recruited to the chromatin in brown adipose tissue via its DNA-binding partners, including C/EBPβ and PPARγ. Notably, PRDM16 deficiency does not compromise the DNA-binding capacity of these partners to their respective target sites; yet the presence of MED1 at these locations is reduced. Moreover, silencing *Med1* in mature brown adipocytes results in a marked decrease in the transcription of genes specific to brown fat. Through its zinc finger domains, PRDM16 directly engages with MED1, positioning it as a super-enhancer for these genes. Furthermore, this interaction is critical for the formation of complex chromatin structures at key gene loci specific to brown adipose tissue (57, 58).

Tyrosine kinase 2 (Tyk2) is a member of the Jak kinase family. Numerous cytokines and growth factors activate the Jak/STAT pathway to stimulate tyrosine phosphorylation of the STAT transcription factors, thereby inducing the expression of early response genes (59). Mice lacking *Tyk2* become progressively obese and exhibit abnormal BAT morphology and function. Constitutively active Stat3 (CAStat3) and PRDM16 restore the differentiation of *Tyk2* KO brown adipocytes. Furthermore, altered BAT morphology and function were restored, and obesity was reversed in *Tyk2* KO mice expressing CAStat3 in BAT. Interestingly, a mutated form of *Tyk2*, which lacks tyrosine kinase activity (Tyk2KD), also rescues the differentiation of T*yk2* KO brown preadipocytes *in vitro*. It is important to note that *Tyk2* KO mice expressing a Tyk2KD transgene in BAT display normal brown adipocyte differentiation and resist the obese phenotype of Tyk KO mice. Mechanistically, STAT3 binds to PRDM16 and enhances the stability of the PRDM16 protein, thereby maintaining brown adipocyte differentiation. Both Tyk2WT and Tyk2KD can also interact with PRDM16 and PGC1α to regulate brown fat differentiation (60, 61).

### 3.3 Co-factors to positively regulate functions of thermogenic adipocytes

High-throughput screening using the *Ucp1* promoter revealed that ZFP516 is a transcriptional activator of *Ucp1*. ZFP516 directly binds to the proximal region of the *Ucp1* promoter to activate *Ucp1* expression by interacting with PRDM16. *Zfp516* is induced by cold and sympathetic stimulation through the cAMP-CREB/ATF2 pathway. Ectopic expression of *Zfp516* represses the myogenic program and enhances brown adipocyte differentiation. Although ablation of *Zfp516* is embryonically lethal, BAT mass in KO embryos is still drastically reduced. Overexpression of *Zfp516* in adipose tissue (aP2-*Zfp516*) promotes the browning of iWAT, increases body temperature and energy expenditure, and prevents diet-induced obesity (62).

LSD1, also known as KDM1A, demethylates the monomethylated or dimethylated histone H3 Lys4 (H3K4me1 or H3K4me2), which are histone markers closely associated with active transcription (63). Quantitative mass spectrometry is used to identify LSD1 from nuclear extracts of primary brown adipocytes with a PRDM16-specific antibody. The brown adipocytes from Adipo-*Lsd1*-KO mice exhibited increased expression of white-selective genes, a blunted response to norepinephrine, and reduced flavin adenine dinucleotide (FAD) levels. It is important to note that Adipo-*Lsd1*-KO mice have more fat mass and lower energy expenditure levels. In line with this result, Ucp1-*Lsd1*-KO mice also gained more body weight on a high-fat diet than control littermates. LSD1 regulates the metabolism of brown adipocytes by cooperating with PRDM16 and ZFP516 (64–66).

Transcription factor Hlx is highly expressed in BAT and iWAT. It is translationally upregulated by β3-adrenergic signaling in iWAT. The knockdown of *Hlx* in BAT and iWAT adipocytes had no effect on adipogenesis but decreased *Ucp1* expression and strongly suppressed oxygen consumption. *Hlx* homozygous null mice are embryonically lethal. *Hlx* heterozygous KO mice exhibit defects in beige adipocyte formation in iWAT and develop glucose intolerance and high-fat-induced hepatic steatosis. Conversely, transgenic expression of *Hlx* promotes the browning of white adipocytes, improves glucose homeostasis, and prevents obesity and hepatic steatosis. Hlx directly interacts with PRDM16 to control BAT-selective gene expression and mitochondrial biogenesis (67).

Whole transcriptome deep sequencing (RNA-seq) analysis of iWAT after cold stimulation identified transcription factor SOX4 as a new regulator of white adipocyte browning. Mice with either Adiponectin-Cre or Ucp1-Cre deletion of *Sox4* exhibited significant cold intolerance, decreased energy expenditure, and impaired beige adipocyte formation. These mice also developed obesity, severe hepatic steatosis, insulin resistance, and inflammation after being fed on a high-fat diet. ShRNA knockdown of *Sox4* in beige adipocytes reduced energy metabolism and the expression of thermogenic genes. SOX4 recruits PRDM16 to PPARγ, thereby forming a transcriptional complex and elevating the expression of thermogenic genes (68).

YAP and TAZ are key molecular effectors of the Hippo pathway in mammals (69). YAP/TAZ acts as a PPARγ co-repressor that regulates adipocyte differentiation. They are required for adipocyte survival in diet-induced obesity and promote obesity-related adipose tissue fibrosis (70, 71). Adipocyte-specific KO of *Yap*/*Taz* (Yap^fl/fl^; Taz^fl/fl^ with AdipoqCre mice) induces S100b expression, thereby stimulating sympathetic innervation and biogenesis of functional beige fat in both iWAT and visceral WAT. S100b is a neurotrophic factor that stimulates the sympathetic innervation of thermogenic fat (72). Calsyntenin 3β was initially suggested to enhance the secretion of S100b in adipocytes, as indicated in reference (72). However, contrasting findings were reported in a subsequent study, which demonstrated that Calsyntenin 3β does not regulate S100b secretion and innervation, as detailed in reference (73). YAP/TAZ competes with C/EBPβ for binding sites on the ZF-2 domain of PRDM16 to inhibit S100b transcription. YAP/TAZ disrupts the formation of the PRDM16-C/EBPβ complex, thereby acting as a brake on beige fat innervation (74).

GTF2IRD1, a cold-inducible and BAT-enriched transcriptional factor, interacts with both PRDM16 and EHMT1 to form a transcriptional complex that represses adipose tissue fibrosis in an UCP1-independent manner. Repressed adipose tissue fibrosis was observed in transgenic mice, where variant 5 of *Gtf2ird1* is driven by the aP2 promoter/enhancer in adipose tissues. This repression was accompanied by improved systemic glucose homeostasis, independent of body weight loss. Deleting *Gtf2ird1* promoted fibrosis in a cell-autonomous manner. GTF2IRD1 represses the transcription of transforming growth factor β-dependent pro-fibrosis genes by recruiting PRDM16 and EHMT1 to their promoter/enhancer regions. Additionally, GTF2IRD1 expression inversely correlates with human subcutaneous WAT fibrosis. The EHMT1-PRDM16-GTF2IRD1 complex plays a crucial role in repressing obesity-associated adipose tissue fibrosis and enhancing systemic glucose homeostasis (75).

### 3.4 Co-factors to negatively regulate functions of thermogenic adipocytes

Transducin-like enhancer protein 3 (TLE3) is an adipogenic coregulator that functions synergistically with PPARγ to stimulate adipogenesis (76). TLE3 counteracts the brown fat program by competing with PRDM16 for interaction with PPARγ. TLE3 and PRDM16 reciprocally regulate BAT- and WAT-selective gene expression. In aP2-*Tle3* transgenic mice, impaired fatty acid oxidation and adipocyte thermogenesis were observed compared to control mice. Conversely, mice lacking *Tle3* in adipose tissue (Adipo-*Tle3* KO) exhibited enhanced thermogenesis in inguinal white adipose depots and were protected from age-dependent deterioration of brown adipose tissue function (77). In humans, TLE3 expression in subcutaneous adipose tissue is increased in type 2 diabetes and decreased following bariatric surgery-induced weight loss (78).

PexRAP synthesizes ether-linked phospholipids that are potential partial agonists for PPARγ and also promotes white adipogenesis (79). Homozygous KO of *PexRAP* in mice resulted in embryonic lethality. To overcome this, Rosa-CreER mice were used to generate tamoxifen-inducible global *PexRAP* KO (*PexRAP*-iKO) animals. In PexRAP-iKO mice, enhanced adipocyte browning, increased energy expenditure, and decreased adiposity were observed. Similar to TLE3, PexRAP disrupts the formation of the PRDM16-PPARγ complex by interacting with PPARγ, thereby inhibiting white adipocyte browning (80).

## 4 Proteins combat obesity by directly regulating Prdm16 expression in adipose tissue

**Figure 3:**
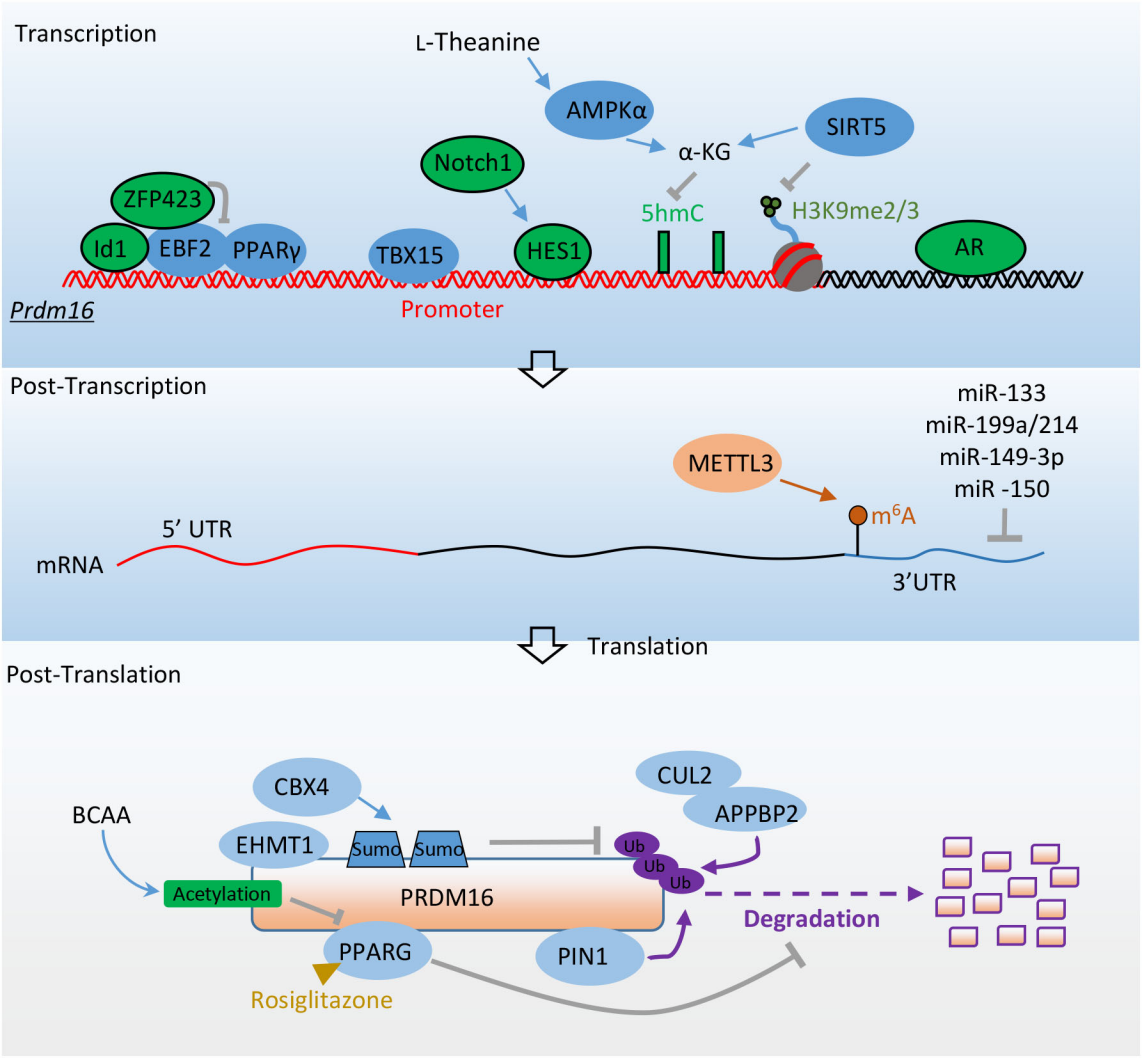
Factors promote adipocyte thermogenesis and resist obesity by directly regulating *Prdm16*’s expression. Transcriptional factors EBF2, PPARγ, and TBX15 promote the transcription of *Prdm16* mRNA. ZFP423 and ID1 inhibit *Prdm16* mRNA transcription by binding with EBF2, while HES1 and AR repress its transcription. AMPKα and SIRT5 enhance *Prdm16* mRNA transcription through epigenetic regulation. At the post-transcriptional level, METTL3 stabilizes *Prdm16* mRNA through m6A modification. Various microRNAs regulate the stability of *Prdm16* mRNA by targeting its 3’ untranslated region (3’UTR). At the post-translational level, the interaction between PPARγ and EHMT1 with PRDM16 enhances its stability. PIN1 promotes the degradation of PRDM16 via the ubiquitin-proteasome system. CBX4 sumoylates PRDM16, preventing its degradation. The CUL2–APPBP2 complex reduces PRDM16 protein stability by catalyzing its polyubiquitination. BCAA promotes the acetylation of PRDM16, disrupting its interaction with PPARγ. More detailed information about these factors is provided in **Table 2**.

The amounts and stabilization of PRDM16 protein are critical in controlling the formation and function of thermogenic adipocytes and in combating obesity. The upstream regulation of PRDM16 has been extensively investigated at both transcriptional and post-transcriptional levels (**Figure 3**; **Table 2**).

**Table 2:** Regulatory factors on the expression of PRDM16.

Protein name	Cellular Function	Physiological Fucntion	Human study	Reference
EBF2	Recruits PPARγ to its brown-selective binding sites including Prdm16.	*Ebf2* overexpression increases mitochondrial function and protect animals against weight gain.	SNP (rs10503776) in exon 11 of *EBF2* is highly associated with an increase in mean BMI in humans.	(46, 85)
TBX15	Bound directly to the *Prdm16* promoter to promote its transcription.	*Tbx15* AKO mice displayed increased body weight gain and decreased insulin senstivity.	*TBX15* expression is strongly down-regulated in visceral adipose depots of obese humans.	(87, 90, 91)
AR	Directly binds within the intronic region of *Prdm16* locus to reduce its expression	Adipose-specific knockout of *Ar* and WT mice showed similar food intake and body weight	*PRDM16* mRNA expression in human WAT of female individuals exhibits increased expression than males.	(94)
Notch1	Notch target Hes1 can bind directly to the promoters of *Prdm16* to repress its xpression.	Notch mutants improves insulin sensitivity and resists high fat diet–induced obesity	None	(96)
AMPKα	Increases DNA demethylation in the Prdm16 promoter to promote its expression.	AICAR, an AMPK acitivator, reduces visceral fat weight in mice.	None	(101, 102)
SIRT5	Decreases H3K9me2 and H3K9me3 levels at promoter regions of *Prdm16* to increase its expression.	*Sirt5* KO mice exhibited more fat mass and impaired glucose tolerance.	None	(103)
METTL3	Decreases m6A modification and expression of Prdm16.	BAT-specific deletion of *Mettl3* promotes obesity and systemic insulin resistance	None	(110)
CBX4	Sumoylates PRDM16 at Lys 917 to block its ubiquitination and augments its stability	Heterozygous mice has significantly increased iWAT mass and deteriorated glucose tolerance.	None	(120)
PIN1	Binds to and promotes the degradation of PRDM16.	Deficiency of *Pin1* in adipocytes protects against obesity, hepatic steatosis, and insulin resistance.	None	(124)
CUL2/APPBP2	Determines PRDM16 protein stability by catalysing its polyubiquitination.	Adipocyte-specifc deletion of *Cul2*–*Appbp2* counteracts obesity, insulin resistance and dyslipidaemia.	*APPBP2* gene is associated with waist–hip ratio adjusted for body mass index	(125)
BCAT2	Acetylates PRDM16 at K915 to disrupt the interaction between PRDM16 and PPARγ	Loss of *Bcat2* in adipocytes protects mice against obesity and insulin resistance	Cold-induced BAT thermogenesis promotes systemic BCAA clearance in humans.	(126)

### 4.1 Transcriptional level regulation

Inhibitor of differentiation 1 (Id1) is a helix-loop-helix transcription factor with a significant role in cell proliferation and differentiation (81). In aP2 promoter-driven *Id1* transgenic mice, there was a propensity for high-fat diet-induced obesity, along with reduced BAT thermogenesis and energy expenditure. *Id1* suppresses BAT thermogenesis by binding to PGC1α and repressing its transcriptional activity. The absence of *Id1* enhances the expression of beige genes in WAT. Additionally, the differentiation of thermogenic genes is increased in embryonic fibroblasts of *Id1*-deficient mice. At the molecular level, Id1 directly binds to EBF2 and suppresses its transcriptional activity, leading to the down-regulation of *Prdm16* expression (82).

The zinc finger transcriptional coregulator 423 (ZFP423) is a transcriptional factor crucial for preadipocyte determination (83). It suppresses the fate of thermogenic adipocytes, with *Zfp423* mRNA being significantly more abundant in white adipocytes compared to brown adipocytes. Inducible deletion of *Zfp423* in the adipocytes of adult mice leads to the accumulation of beige adipocytes in the iWAT depot. This loss of *Zfp423* is associated with increased energy expenditure and relatively lower diet-induced weight gain. Conversely, overexpression of *Zfp423* converts BAT to a more unilocular, white adipocyte-like phenotype, suppressing the expression of thermogenic genes. *Zfp423*-deficient adipocytes can be converted to beige adipocytes in a cell-autonomous manner. ZFP423 recruits the NuRD corepressor complex to EBF2-bound thermogenic gene enhancers, including *Prdm16.* Conversely, deletion of *Zfp423* induces a coregulator switch from the NuRD complex to the BAF complex on EBF2, resulting in a shift in PPARγ occupancy to thermogenic gene enhancers (84, 85).

T-box 15 (Tbx15) belongs to a phylogenetically conserved family, which has a similar characteristic sequence in the DNA binding domain and is involved in various developmental processes (86). *Tbx15* is highly expressed in brown adipose tissue and iWAT, minimally expressed in visceral white adipose tissue. In mice, siRNA knockdown of *Tbx15* in brown and inguinal adipose stromal cells impaired their differentiation into mature adipocytes (87). Paradoxically, overexpression of *Tbx15* in 3T3-L1 preadipocytes blunted their differentiation into adipocytes (88). *In vivo*, the heterozygous deletion of *Tbx15* resulted in glucose intolerance and obesity when the mice were fed on high-fat diets (89). Furthermore, Adipo-*Tbx15* KO mice displayed increased body weight gain and decreased whole-body energy expenditure in response to high-fat diets. In humans, *TBX15* expression is strongly downregulated in visceral adipose depots of overweight and obese individuals (90). TBX15 could directly bind to the *Prdm16* promoter to enhance its transcription and participate in adipocyte browning induced by the adrenergic signaling pathway (91).

Androgens play a crucial role in regulating body fat distribution and function. Their excess is significantly correlated with body mass index, abdominal obesity, and insulin resistance (92, 93). Using the *Prdm16* luciferase knock-in reporter mouse model, it has been observed that the androgen receptor (AR) shows the strongest negative correlation with *Prdm16*. Activation of androgen-AR signaling suppresses *Prdm16* expression and reduces the beiging process in beige adipocytes. Adipocyte-selective ablation of *Ar* promotes beige adipogenesis, whereas its adipocyte-specific overexpression inhibits the browning of white adipocytes. A sex dimorphism in *Prdm16* mRNA expression is also evident in human WAT, with female individuals exhibiting higher expression levels than males. Additionally, an analysis of a recent single-nuclei RNA sequencing dataset from human visceral adipose tissue indicates a trend toward increased *PRDM16* expression in female adipocytes compared to male adipocytes (49). ChIP analysis reveals that AR directly binds to the intronic region of the *Prdm16* locus, negatively regulating its expression (94).

Notch receptors (Notch1-Notch4) are activated by binding to Dll or Jag family ligands, followed by cleavage to release the Notch intracellular domain (NICD). NICD then binds with the Rbpj transcriptional complex to activate the transcription of downstream targets, including *Hes* (95). Adipose-specific inactivation of Notch1 triggers the browning of white adipocytes and elevates the expression of thermogenic genes. In contrast, adipose-specific activation of Notch1 (N1ICD) inhibits the browning of white adipocytes and is associated with higher expression levels of *Hes1* and *Hey1* compared to WT mice. Overexpression of the transcriptional repressor *Hes1* in adipose stromal cells of WT mice results in lower levels of *Prdm16* and *Pgc1α*. At the molecular level, the Notch target HES1 can directly bind to the promoters of *Prdm16* and *Pgc1α*, repressing their expression and thereby inhibiting thermogenic gene programs (96).

AMP-activated protein kinase (AMPK) indirectly regulates BAT thermogenic function by influencing the hypothalamus and the adrenergic nervous system (97). It directly regulates brown adipogenesis by elevating α-ketoglutarate (αKG) production and promoting DNA demethylation in the *Prdm16* promoter of progenitor cells. *Prdm16* is enriched with CpG sites surrounding its transcription start site (TSS) (98). Active DNA demethylation, essential for initiating its expression, is mediated by the ten-eleven translocation hydroxylases (TETs) (99). The TET catalytic reaction requires αKG, a key metabolite of the Krebs cycle, thereby linking metabolism to epigenetic modifications (100). Ablation of *AMPKα1* reduces the activity of isocitrate dehydrogenase 2 and cellular αKG levels, leading to an increase in 5-methylcytosine (5mC) and a decrease in 5-hydroxymethylcytosine (5hmC) in the *Prdm16* promoter. Consequently, *AMPKα1* knockout suppresses brown adipogenesis, while its activation promotes brown adipogenesis (101). L-theanine, a non-proteinogenic amino acid, enhances AMPKα phosphorylation, to increase αKG levels and to activate DNA demethylation on the *Prdm16* promoter. When L-theanine was administered to high-fat diet-fed mice, it resulted in increased energy expenditure, reduced obesity, and improved glucose tolerance and insulin sensitivity (102).

SIRT5, a member of the sirtuin family, is essential for brown adipocyte differentiation and the expression of brown adipogenic genes. Knockdown of *Sirt5* reduces intracellular αKG concentrations while elevating H3K9me2 and H3K9me3 levels at the promoter regions of *Pparγ* and *Prdm16* loci. This inhibits the expression of *Pparγ* and *Prdm16*, thereby impairing adipocyte thermogenesis. *Sirt5* deficiency in mice reduces the browning capacity in iWAT and leads to insulin resistance and obesity (103). Other sirtuins contribute to adipocyte function by modulating other genes. For example, SIRT1 facilitates the browning of white adipose tissue by deacetylating PPARγ (104). SIRT2 impedes adipocyte differentiation by deacetylating FOXO1 (105). SIRT3 is known to regulate mitochondrial function and thermogenesis in brown adipocytes, enhancing the expression of PGC1α (106).

### 4.2 Posttranscriptional level regulation

Methyltransferase-like 3 (METTL3), a key RNA methyltransferase, is highly expressed in BAT and plays an essential role in postnatal BAT development and maturation. RNA m6A modification is one of the most prevalent mRNA modifications in eukaryotes. It can be catalyzed by m6A writer proteins such as METTL3, recognized by m6A reader proteins, and removed by m6A eraser proteins (107). RNA m6A modification regulates most RNA processing steps, including mRNA splicing, stability, and translation efficiency, which further modulate other biological processes (108, 109). Deletion of *Mettl3* driven by the Ucp1 promoter severely impairs the maturation of BAT by reducing m6A peaks and the expression of *Prdm16*, *Pparγ*, and *Ucp1*. This significantly reduces BAT-mediated adaptive thermogenesis and promotes high-fat diet-induced obesity and systemic insulin resistance (110).

Non-coding strands of RNA, including microRNAs, regulate gene expression at the transcriptional level (111). *Prdm16* mRNA expression is controlled by several miRNAs, including miR-133 and miR-199a/214, which directly target the 3’ untranslated region (3’ UTR) of *Prdm16* and negatively regulate its expression. These microRNAs are potential therapeutic targets for inducing brown or beige adipocyte lineage differentiation, thereby increasing energy expenditure and reducing fat mass in obese individuals. Inhibition of miR-133 leads to the differentiation of precursors in BAT and skeletal muscle stem cells into mature brown adipocytes, increasing whole-body energy expenditure, improving glucose tolerance, and impeding the development of diet-induced obesity (112, 113). Conversely, overexpression of the miR-199a/214 cluster suppresses the differentiation of brown adipocytes, while knockdown of this cluster increases thermogenic gene expression in beige adipocytes (114). Overexpression of miR-149-3p promotes a visceral-like switch during inguinal preadipocyte differentiation (115, 116). The KH-type splicing regulatory protein (KSRP), an RNA-binding protein, enhances the expression of miR-150 in iWAT, attenuating the expression of brown fat genes (117). Taken together, *Prdm16* mRNA expression is regulated by various microRNAs to facilitate the expression of brown-selective genes and resist obesity.

### 4.3 Post-translational level regulation

PRDM16 protein is short-lived and rapidly degraded, making the increase of its lifetime in adipose tissue a promising strategy for combating obesity. Several studies have shed light on the regulation of PRDM16 protein stabilization in adipose tissue.

The full agonist of PPARγ, rosiglitazone, induces the expression of brown fat genes such as *Ucp1* in primary adipocytes differentiated from the stromal-vascular fraction (SVF) of iWAT, but not in those from epididymal WAT. *Prdm16* mRNA expression is significantly higher in iWAT than in epididymal WAT. ShRNA knockdown of *Prdm16* blunts the effects of the PPARγ agonist-induced browning of inguinal white adipocytes. Conversely, transgenic expression of *Prdm16*, together with rosiglitazone, synergistically induces the expression of thermogenic genes and the emergence of UCP1-positive multilocular adipocytes *in vivo*. The white-to-brown fat conversion induced by rosiglitazone is partly regulated through the enhanced stability of PRDM16 protein and inhibition of the ubiquitin-proteasome pathway (118).

CBX4, a Polycomb group protein, acts as a SUMO E3 ligase for PRDM16 and mediates its sumoylation. The expression of *Cbx4* is enriched in brown adipose tissue and is induced by acute cold exposure. CBX4 sumoylates PRDM16 at lysine 917, preventing its ubiquitination-mediated degradation and enhancing its stability mediated by EHMT1. Homozygous *Cbx4* knockout mice die almost immediately after birth (119). Heterozygous *Cbx4* knockout reduces PRDM16 protein levels, impairs white adipocyte browning, significantly increases iWAT mass, and deteriorates glucose tolerance and cold tolerance. Furthermore, fat-specific *Cbx4* knockdown or overexpression through adenovirus injection remodels iWAT, decreasing or increasing adipocyte thermogenesis by down-regulating or up-regulating PRDM16 protein levels, respectively (120).

Pin1 is a unique enzyme associated with the phospho-serine or phosphor-threonine-proline-containing motif of numerous substrates where it controls their functions and stability (121). *Pin1* expression in adipose tissue is markedly increased by obesity. As a result, *Pin1* KO mice are highly resistant to diet-induced obesity (122, 123). Adipocyte-specific *Pin1* KO (adipo-*Pin1* KO) promotes thermogenesis, resists high-fat diet-induced obesity, and improves glucose tolerance. At the molecular level, PIN1 binds to PRDM16 and promotes its degradation through the ubiquitin-proteasome system, suppressing non-shivering thermogenesis (124).

Cullin–RING member 2 (CUL2) functions as a scaffold protein by interacting with an E2 enzyme, elongin B (ELOB), elongin C (ELOC), and a substrate-specific receptor (amyloid precursor protein-binding protein (APPBP2)). APPBP2 directly interacts with PRDM16. CUL2–APPBP2 is an ubiquitin E3 ligase that determines PRDM16 protein stability by catalyzing its polyubiquitination, without altering its mRNA expression. Inhibiting CUL2–APPBP2 sufficiently extended the half-life of the PRDM16 protein and promoted the biogenesis of beige adipocytes. Adipocyte-specific deletion of *Cul2* or *Appbp2* counteracted diet-induced obesity, glucose intolerance, and insulin resistance while promoting adipocyte thermogenesis (125).

Branched-chain amino acids (BCAAs), including leucine, isoleucine, and valine, are converted to branched-chain keto acids (BCKAs) by BCAT1 (BCATc, cytosolic branched-chain aminotransferase) or BCAT2 (BCATm, mitochondrial branched-chain aminotransferase). Adipose tissue *Bcat2* KO mice exhibit increased iWAT browning and thermogenesis, and have an increased ability to resist high-fat diet-induced obesity. BCKA supplementation rescues *Bcat2* KO mice from high-fat diet-induced obesity. Moreover, telmisartan, an anti-hypertension drug and direct inhibitor of BCAT2, enhances WAT browning and reduces adiposity. Mechanistically, acetyl-CoA derived from BCKA promotes acetylation of PRDM16 at K915, thereby disrupting its interaction with PPARγ. This prevents iWAT browning and BAT thermogenesis (126, 127). Furthermore, BAT actively utilizes BCAA in the mitochondria for thermogenesis and enhances systemic BCAA clearance in both mice and humans upon cold exposure. A BAT-specific defect in BCAA catabolism can attenuate systemic BCAA clearance and thermogenesis, which lead to diet-induced obesity and glucose intolerance (126). Additionally, BAT catabolize BCAA in the mitochondria, serving as nitrogen donors for the biosynthesis of non-essential amino acids and glutathione. This process which helps to reduce oxidative stress and increase hepatic insulin signaling is independent of BAT thermogenesis (128).

## 5 Discussion and prospectives

The activity and expression of PRDM16 in adipose tissue are closely associated with adipocyte thermogenesis and resistance to obesity, as evidenced by these distinguished studies. Consequently, directly targeting PRDM16 proteins in thermogenic adipose tissue emerges as a potential strategy for combating obesity and related metabolic disorders. Interventions that increase the expression of PRDM16, reduce its degradation, or stabilize the PRDM16 protein could enhance the formation and function of brown and beige adipocytes. Numerous studies have reported that various drugs can alleviate obesity and diabetes by modulating the expression and function of PRDM16. For example, resveratrol is known to activate adipocyte thermogenesis and counteract obesity induced by a high-fat diet by upregulating the expression of PRDM16 (129). Similarly, rutaecarpine has been shown to promote adipocyte thermogenesis via the AMPK-PRDM16 signaling pathway (130). A comprehensive summary of studies focusing on drugs targeting PRDM16 is available in another review (131).

Humans with active BAT exhibit a healthier body fat distribution, characterized by reduced visceral adipose tissue and increased subcutaneous adipose tissue. Additionally, these individuals demonstrate improved metabolic profiles, including lower levels of blood glucose and lipids, as well as decreased liver fat accumulation. A significant association between the presence of BAT and enhanced cardiometabolic health has also been observed in overweight or obese individuals (132, 133). Exposure to mild cold has been shown to activate adrenergic receptor signaling pathways, which in turn enhance glucose and fatty acid uptake, as well as oxidative metabolism within human BAT (134, 135). Oral administration of mirabegron, a β3 adrenergic receptor agonist approved for the treatment of overactive bladder, has the potential to activate human BAT and augment whole-body energy expenditure (136, 137). Despite this, significant weight loss in obese patients following mirabegron treatment has not yet been conclusively demonstrated (138). In human subjects, the most efficacious stimulation of adipose tissue has been observed with higher doses of mirabegron (139). However, potential cardiovascular side effects may restrict the clinical use of such dosages (140).

Obesity arises from an energy imbalance, characterized by excessive energy intake or insufficient energy expenditure. To combat obesity, one approach is to enhance energy expenditure, including the promotion of adipocyte thermogenesis. Another strategy involves reducing energy intake. Glucagon-like peptide-1 (GLP-1) receptor agonists, such as liraglutide and semaglutide, have proven effective in resisting obesity and improving hyperglycemia, insulin sensitivity, and blood pressure by inhibiting food intake, as demonstrated in both preclinical and clinical studies. Interestingly, in preclinical experiments, liraglutide and semaglutide have also shown the ability to induce adipocyte thermogenesis, evidenced by elevated expression of UCP1 and PRDM16 proteins and improvements in insulin sensitivity (141, 142). However, the administration of GLP-1 receptor agonists in humans does not appear to significantly contribute to weight loss through increasing adipocyte thermogenesis. Clinical trials have shown that once-daily liraglutide administration in individuals with obesity and type 2 diabetes reduces body weight and decreases resting energy expenditure, but does not alter the proportion of fat within brown adipose tissue (143). Similarly, once-weekly semaglutide administration in individuals with obesity reduced food intake and fat mass without changing resting energy expenditure (144). Therefore, while GLP-1 receptor agonists may promote adipocyte thermogenesis in rodents, their role in this mechanism in humans remains to be clearly defined (145).

To date, no clinically approved drugs effectively increase adipocyte thermogenesis for the treatment of obesity. Compared to β3 adrenergic receptor agonist and GLP-1 receptor agonist, PRDM16 protein could represent a novel and promising therapeutic approach for combating obesity and its associated metabolic disorders. However, unlike these agonists, PRDM16 is not a direct drug target. Therefore, alternative pathways must be modulated to regulate the expression and function of PRDM16 in adipose tissue. Furthermore, *Prdm16* is not exclusively expressed in thermogenic adipocytes like *Ucp1*; it is also expressed in various other tissues including skeletal and cardiac muscle. *Prdm16* has been implicated in the regulation of hematopoietic and neural stem cell maintenance, cardiac development, and other pathological conditions (146–149). In the absence of PRDM16, there is an increase in reactive oxygen species and apoptosis within hematopoietic stem cells. Global mutations in PRDM16 are associated with cleft palate, altered craniofacial development, and impaired cardiac development. In humans, PRDM16 mutations have been identified as a cause of cardiomyopathy. Brown adipocytes share a common precursor with myocytes, and PRDM16 is a pivotal effector in the conversion of myoblasts into brown adipocytes. Therefore, further studies are warranted to examine the potential effects of drugs targeted at PRDM16 on muscle development and function *in vivo*. In summary, it is still a long way to combat obesity and its related metabolic disorders by targeting PRDM16 protein in thermogenic adipose tissue.
